# Comparison of conventional and Si-photomultiplier-based PET systems for image quality and diagnostic performance

**DOI:** 10.1186/s12880-019-0377-6

**Published:** 2019-10-22

**Authors:** Jenny Oddstig, Sigrid Leide Svegborn, Helen Almquist, Ulrika Bitzén, Sabine Garpered, Fredrik Hedeer, Cecilia Hindorf, Jonas Jögi, Lena Jönsson, David Minarik, Richard Petersson, Annika Welinder, Per Wollmer, Elin Trägårdh

**Affiliations:** 1 Radiation Physics, Skåne University Hospital and Lund University, Carl Bertil Laurells gata 9, 20502 Malmö and Lund, Sverige; 2 Clinical Physiology and Nuclear Medicine, Skåne University Hospital, 221 85 Lund, Sweden; 3 Clinical Physiology and Nuclear Medicine, Skåne University Hospital and Lund University, Carl Bertil Laurells gata 9, 20502 Malmö, Sverige; 4 Wallenberg Centre for Molecular Medicine, Lund University, Lund, Sweden

**Keywords:** PET-CT, FDG, Digital PET, Image quality, Oncology

## Abstract

**Background:**

A new generation of positron emission tomography with computed tomography (PET-CT) was recently introduced using silicon (Si) photomultiplier (PM)-based technology. Our aim was to compare the image quality and diagnostic performance of a SiPM-based PET-CT (Discovery MI; GE Healthcare, Milwaukee, WI, USA) with a time-of-flight PET-CT scanner with a conventional PM detector (Gemini TF; Philips Healthcare, Cleveland, OH, USA), including reconstruction algorithms per vendor’s recommendations.

**Methods:**

Imaging of the National Electrical Manufacturers Association IEC body phantom and 16 patients was carried out using 1.5 min/bed for the Discovery MI PET-CT and 2 min/bed for the Gemini TF PET-CT. Images were analysed for recovery coefficients for the phantom, signal-to-noise ratio in the liver, standardized uptake values (SUV) in lesions, number of lesions and metabolic TNM classifications in patients.

**Results:**

In phantom, the correct (> 90%) activity level was measured for spheres ≥17 mm for Discovery MI, whereas the Gemini TF reached a correct measured activity level for the 37-mm sphere. In patient studies, metabolic TNM classification was worse using images obtained from the Discovery MI compared those obtained from the Gemini TF in 4 of 15 patients. A trend toward more malignant, inflammatory and unclear lesions was found using images acquired with the Discovery MI compared with the Gemini TF, but this was not statistically significant. Lesion-to-blood-pool SUV ratios were significantly higher in images from the Discovery MI compared with the Gemini TF for lesions smaller than 1 cm (*p* < 0.001), but this was not the case for larger lesions (*p* = 0.053). The signal-to-noise ratio in the liver was similar between platforms (*p* = 0.52). Also, shorter acquisition times were possible using the Discovery MI, with preserved signal-to-noise ratio in the liver.

**Conclusions:**

Image quality was better with Discovery MI compared to conventional Gemini TF. Although no gold standard was available, the results indicate that the new PET-CT generation will provide potentially better diagnostic performance.

## Background

Positron emission tomography with computed tomography (PET-CT) is a well-established and fast-growing imaging modality, mainly used in oncology [1]. Over the years, improvements in detector design as well as the implementation of time-of-flight technology and better reconstruction methods have significantly improved image quality and introduced the possibility of reducing the amount of activity administered and/or scanning time [2]. A new generation of PET-CT scanners has recently been introduced, and they use silicon (Si) photomultiplier (PM)-based technology. This technology has the potential to increase the detection of pathology, primarily through greater sensitivity. It is hoped that this improved detectability will enable earlier detection of pathologies, including metastatic spread. Thus far, this remains to be confirmed in patient studies. Using the National Electrical Manufacturers Association (NEMA) NU-22012 standard [3], Hsu et al. [4] found that SiPM-based PET cameras have greater sensitivity and time resolution compared to conventional PM-based PET cameras. They also presented a clinical case where metastases were detected using the SiPM-based PET camera but not using a conventional PM-based PET camera. Two other studies comparing SiPM-based PET-CT to conventional PET-CT found the SiPM-based PET-CT performed better and detected a greater number of lesions [5, 6].

The aim of this study was to compare the quality and diagnostic performance of images obtained from a novel SiPM-based PET system (Discovery MI; GE Healthcare, Milwaukee, WI, USA), using the Bayesian penalized-likelihood reconstruction algorithm (Q.Clear), with those obtained from a conventional PM-based PET system with time-of-flight (Gemini TF; Philips Healthcare, Cleveland, OH, USA), using the line-of-response row-action maximum-likelihood algorithm, in phantom and patients undergoing clinical PET-CT scanning with ^18^F-FDG. Thus, images from two PET-CT cameras with reconstruction algorithms per vendor’s recommendations were compared; i.e. clinically relevant protocols were evaluated.

## Methods

### The PET-CT systems

The imaging of the phantom and the patients was carried out on two different PET-CT systems, a GE Discovery MI installed 2017 and a Philips Gemini TF installed 2007, see Table 1 for specifications. Both PET-CT scanners are cross-calibrated to the activity meter, and the calibration is validated monthly in a standardized uptake value (SUV) control of phantoms.

**Table 1:** Characteristics of the Discovery MI and Gemini TF PET-CT systems

PET-CT system	Discovery MI	Gemini TF
Crystal material	LYSO	LYSO
Amplifier	SiPM	PM-tubes
Number of rings	4	3
Size of crystals [mm^3^]	3.95 × 5.3 × 25	4.0 × 4.0 × 22
Axial FOV [mm]	200	180
Bore diameter [mm]	700	700
Overlap [%]	23.9	50
NEMA Sensitivity [cps/kBq]	13.8	7.0
FWHM axial @1 cm [mm]	4.7	5.8
Coincidence window width (ns)	4.9	6
Timing resolution (ps)	382	600
Lower energy threshold (keV)	425	440
Matrix	256 × 256	144 × 144
Pixel size (mm^2^)	2.7 × 2.7	4.0 × 4.0
Slice thickness [mm]	2.79	4.0
Image planes in the axial FOV	71	45
Number of CT slice	128	16

*FOV* field of view; *FWHM* full width at half maximum; *LYSO* lutetium-yttrium oxyorthosilicate; *NEMA* National Electrical Manufacturers Association; *PM* photomultiplier; *SiPM* silicon photomultiplier

### Phantom studies

The NEMA IEC Body Phantom (Data Spectrum, Durham, NC, USA) with fillable spheres (10, 13, 17, 22, 28 and 37 mm diameter) and a cylindrical lung insert was used. Its background volume was filled with an ^18^F-FDG concentration of 4.7 ± 0.1 kBq/mL for the Discovery MI and 4.9 ± 0.1 kBq/mL for the Gemini TF. To yield a sphere-to-background activity ratio of four, activity concentrations of 18.8 ± 0.4 kBq/mL and 18.7 ± 0.4 kBq/mL, respectively, were used for the spheres. The phantom was scanned in one bed position using the clinical parameters on each camera, which were 1.5 min/bed position, for the Discovery MI and 2 min/bed position, for the Gemini TF. Acquisition was carried out in list mode with time-of-flight enabled for both systems. No motion-correction was performed. All CT imaging was performed using a low-dose CT protocol at 120 kV, 30–160 mA and a noise index of 45 for the Discovery MI and at 120 kV and 50 mA without tube current modulation for the Gemini TF. Image reconstructions were performed according to routine clinical practice with reconstruction parameters to obtain the best clinical image per system. For the Discovery MI, reconstruction was performed using a Bayesian penalized-likelihood reconstruction algorithm (Q.Clear) [7] with a beta value of 550, using time of flight and the point spread function modelling for recovery of the resolution. For the Gemini TF, the line-of-response row-action maximum-likelihood algorithm method, the so-called BLOB-OS-TOF was used, with time of flight but without the point spread function over 3 iterations, 33 subsets and a 5-mm Gaussian post-filter.

In the image corresponding to the central slice of spheres, regions of interests (ROIs) were drawn along the inside borders of the spheres in the CT image, and this was copied onto the PET images. Mean activity concentrations in spheres and backgrounds were then measured. The recovery coefficient (RC) was calculated as the ratio between the measured and the true activity concentration.

### Patient studies

#### Patients

Patients aged 18 years or more who were referred for clinical ^18^F-FDG-PET-CT to Skåne University Hospital, Malmö between May 82,017 and May 232,017 were invited to participate in the study. A total of 16 patients were included. This study was approved by the Regional Ethical Review Board (#2016/417) and the Radiation Protection Committee (#SSFo2016–018) and was performed in accordance with the Declaration of Helsinki. All patients gave written consent.

#### Imaging

All patients underwent a single intravenous injection of ^18^F-FDG (4 MBq/kg) after fasting for at least 4 h and a dual-imaging protocol on the Discovery MI and Gemini TF PET-CT scanners. Of the 16 patients, nine were first imaged on the Gemini TF, and seven were first imaged on the Discovery MI. For the first acquisition, we aimed for a 60-min accumulation time, as per standard practice in our clinic. Images were acquired from the inguinal region to the base of the skull (1.5 min per bed position for the Discovery MI and 2 min per bed position for the Gemini TF). The images were reconstructed as described above. No gating for respiratory motion was applied.

CT imaging was acquired for attenuation correction and anatomic correlation of the PET images. According to our clinical routine, a diagnostic CT with intravenous contrast was performed if no previous diagnostic CT had been performed within 4 to 6 weeks. In 9 patients, a diagnostic CT was performed with the first PET acquisition, and a low-dose CT was performed with the second PET acquisition. In two patients, the opposite order was applied. In the remaining five patients, low-dose CT was performed for both PET acquisitions. For diagnostic CTs, tube current modulation was applied (the adaptive statistical iterative reconstruction V-technique, ASiR-V for Discovery MI and DoseRight for Gemini TF), adjusting the tube current for each individual and thus optimising the radiation dose. A tube voltage of 100 kV was used for the Discovery MI and 120 kV for the Gemini TF. The low-dose CT was acquired using the same tube current and voltage as described for the phantom CT acquisitions.

#### Image analysis

##### Image quality

The signal-to-noise ratio (SNR) was calculated from ROIs drawn in liver transaxial images. A 4 cm^2^ ROI was drawn in liver segment 5/6 on images acquired from the Discovery MI, and it was copied to the images acquired from the Gemini TF. The ROIs were drawn in three subsequent transaxial slices, and the measurements were averaged. None of the ROIs were placed where liver metastasis was seen. The SNR was calculated by dividing the SUV_mean_ in the ROI by its standard deviation. The SUVs and lesion-to-blood-pool (blood pool measured in the left atria) SUV ratios were calculated by an experienced nuclear medicine physician. A maximum of six ^18^F-FDG-avid lesions were selected for each patient (the same for both camera systems). If available, malignant lesions were included; otherwise lesions interpreted as inflammatory or unclear were included. Lesions were chosen so that a variety of sizes (< 1 cm and ≥ 1 cm on the short axis measured on transversal CT images) were included. If possible, lesions from a variety of body regions were chosen. The lesion-to-blood-pool ratios were calculated as: lesion SUV_max_/blood pool SUV_mean_.

##### Image interpretation

All images were reviewed on an Extended Brilliance Workspace workstation (Philips Healthcare). Three experienced nuclear medicine physicians together reviewed the two PET datasets. Images were analysed for the number of pathologic lesions and were classified as “malignant/probably malignant lesion”, “inflammatory/probably inflammatory lesion” or “unclear lesion”. Furthermore, a simplified metabolic TNM classification (if applicable) was performed. It was defined as follows: was a primary tumour present or not (T+ or T-), did it spread to lymph nodes or not (N+ or N-) and were distant metastases present or not (M+ or M-). The presence of T+, N+ or M+ was considered a worse stage than T-, N- or M-. Patient images were anonymized and randomized, then given to the experts for interpretation. Image sets were separated by camera, and those acquired using the Gemini TF were interpreted first. The physicians were provided with basic patient information (i.e., sex, age, indication for the examination, and the clinically-written CT report). Where only a low-dose CT was performed together with the PET acquisition, the diagnostic CT performed on the other scanner (or, if a separate diagnostic CT was performed within 4–6 weeks before the PET-CT, according to routine clinical practice), was provided to the interpreting physicians (see Imaging section).

### Statistics

Differences in SNR in the liver, number of lesions and SUV parameters were assessed using paired *t*-tests. Differences in metabolic TNM classification were calculated as 95% confidence intervals (CIs) of a proportion using the modified Wald method. Statistical significance was recognized at *p* ≤ 0.05 unless otherwise specified. Bonferroni adjustments were used to account for multiple comparisons for SUV lesion parameters.

## Results

### Phantom studies

**Fig. 1:**
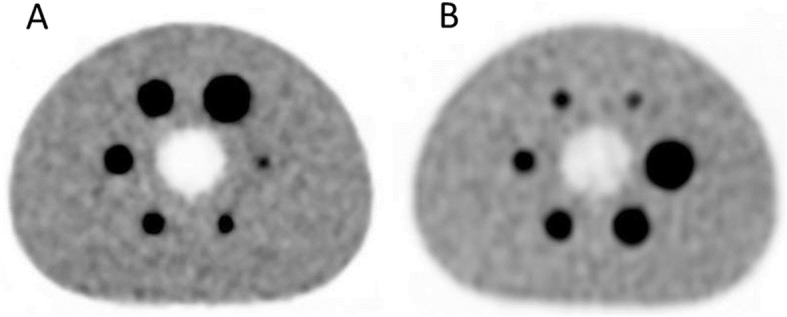
Transversal images of the phantom obtained from **a**) Discovery MI and **b**) Gemini TF with a sphere-to-background activity ratio of 4:1. Image acquisitions and reconstructions were performed as in the patient study

**Fig. 2:**
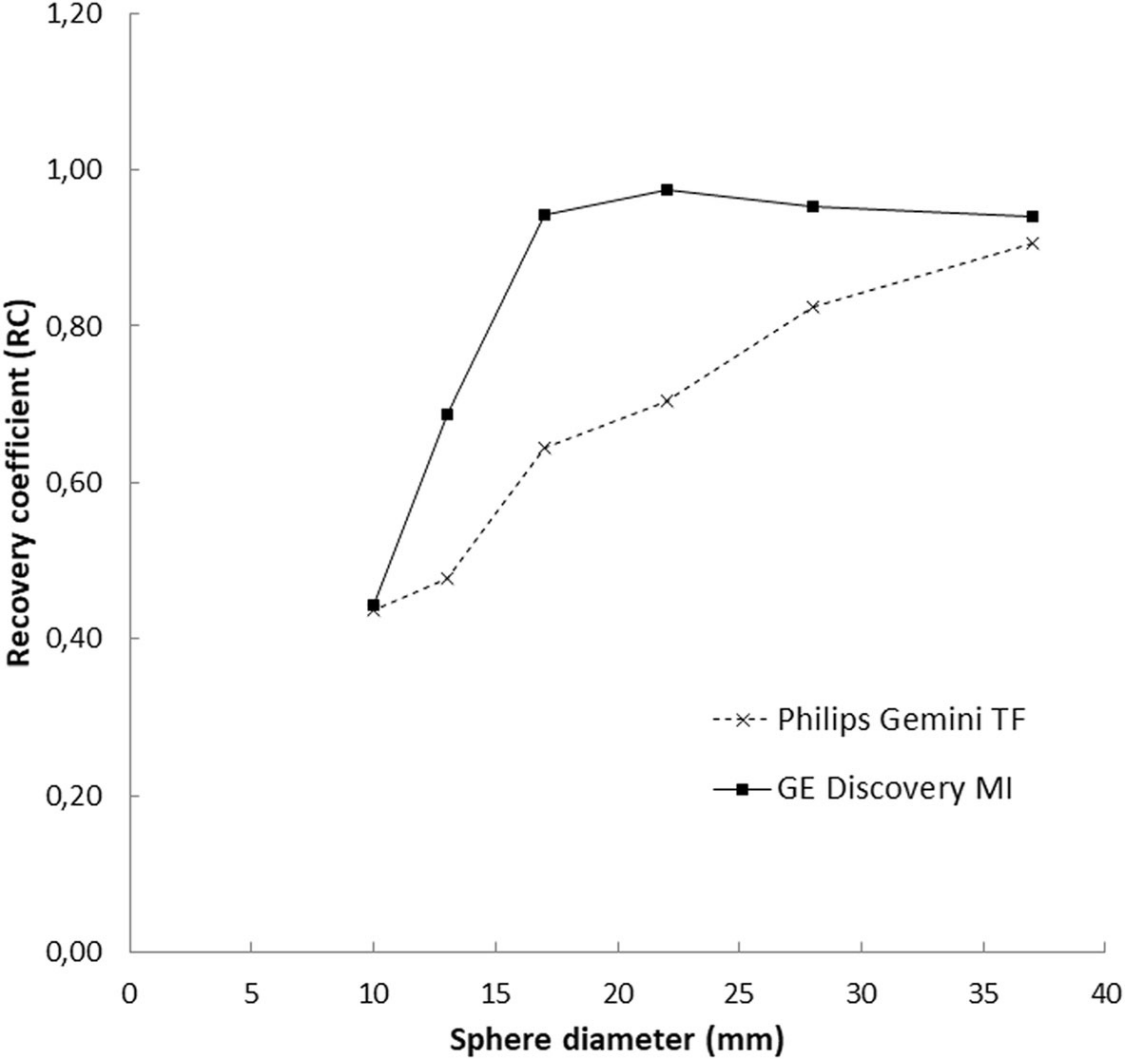
Recovery coefficients for two scanners across various sphere diameters in the phantom with a sphere-to-background activity ratio of 4:1. Scanning acquisition and image reconstruction parameters identical to those used for a clinical patient. Results based on the mean activity concentration achieved from regions of interest drawn on the CT image then copied to the PET image

Figure 1 shows images obtained from the phantom on both systems. Measured mean activity concentration was underestimated by both for the 10-mm diameter spheres. The Discovery MI measured the activity level accurately compared to the activity meter (> 90%) for spheres 17 mm or greater in diameter. As seen in Fig. 2, the underestimation was comparatively larger when using the Gemini TF with spheres measuring 13, 17, 22 or 28 mm in diameter. The Gemini TF reached an activity level close to that measured by the activity meter (> 90%) for the 37-mm spheres. Table 2 shows the activity levels measured for the spheres and backgrounds. The SNRs in the backgrounds were 11.7 and 14.9 for the Discovery MI and Gemini TF, respectively.

**Table 2:** Measured activity concentration in images from the Discovery MI and the Gemini TF compared to the true activity concentration (measured by ionization chamber)

	Discovery MI	Gemini TF
True activity concentration (kBq/ml)	19.64	19.53
Sphere diameter (mm)	Measured activity concentration (kBq/ml)	
37	18.45	17.68
28	18.73	16.08
22	19.13	13.77
17	18.49	12.59
13	13.52	9.34
10	8.69	8.54

### Patient studies

#### Patients

Sixteen (16) patients were included. Patient characteristics and their indications for the PET-CT are shown in Table 3. Mean accumulation time for the nine patients first imaged on the Gemini TF was 66 ± 7 min for the Gemini TF and 103 ± 10 min for the Discovery MI. For those imaged in the reverse order, mean accumulation time was 67 ± 8 min for the Discovery MI and 98 ± 17 min for the Gemini TF. Overall, mean accumulation time was 91 ± 17 min for images obtained with the Discovery MI and 76 ± 22 min for images obtained with the Gemini TF (*p* = 0.04).

**Table 3:** Patient characteristics

No.	Tumour type/Indication	Referral category	Age (yr)	Sex	BMI (kg/m^2^)	Glucose level (mg/dL)	Acc. time Gemini TF (min)	Acc. time Discovery MI (min)
1	Lung cancer	1	70–80	M	26.3	109.8	81	123
2	Malignancy? Vasculitis?	1	60–70	F	24.7	106.2	74	113
3	Pancreatic cancer	2	70–80	F	24.2	108.0	63	102
4	Lung cancer	1	80–90	F	18.4	108.0	63	96
5	Lung cancer	1	70–80	M	28.4	109.8	65	100
6	Malignant melanoma	2	70–80	M	26.6	82.8	64	110
7	Uterus cancer	2	40–50	F	22.4	86.4	59	97
8	Anal cancer	2	40–50	M	25.1	86.4	60	93
9	Pancreatic cancer	2	70–80	F	21.6	122.4	104	61
10	Lung cancer	1	80–90	M	25.9	140.4	118	80
11	Esophageal cancer	1	40–50	M	23.0	113.4	120	76
12	Sarcoidosis	1	40–50	M	26.3	99.0	100	70
13	Uterus cancer	2	70–80	F	17.1	102.6	61	92
14	Lung cancer	1	60–70	F	31.6	100.8	81	62
15	Lung cancer	1	60–70	F	32.7	95.4	79	62
16	Lung cancer	1	60–70	M	23.9	104.4	82	61

*1, initial evaluation/diagnosis/staging; 2, follow-up after treatment. *Acc. time* accumulation time, *BMI* body mass index

#### Image quality and lesion analysis

The mean SNR in the liver was 11.9 ± 3.2 and 12.7 ± 2.7 for the Discovery MI and Gemini TF, respectively. The difference was not statistically significant (*p* = 0.52). SUV_mean_ in the liver was 2.4 for both systems.

In total, 68 lesions were analysed regarding SUV, 32 of which measured smaller than 1 cm. Lesion SUV_max_ was 8.3 ± 6.5 for images from the Discovery MI and 5.2 ± 4.5 for images from the Gemini TF (*p* < 0.001). The SUV_mean_ in the blood pool was 1.7 ± 0.4 in images from the Discovery MI and 1.5 ± 0.3 in images from the Gemini TF (*p* = 0.02). The lesion-to-blood-pool ratio was 4.9 ± 4.0 for images from the Discovery MI and 3.7 ± 3.4 for images from the Gemini TF (*p* < 0.001). Details of lesion SUV parameters and subgroup analysis for all lesions are shown in Table 4. All absolute SUV_max_ values were greater in lesions found using the Discovery MI compared to those found using the Gemini TF, with one exception. For patients first imaged on the Discovery MI, the difference was 2.3 ± 1.9 (*n* = 30), and for those first imaged on the Gemini TF, it was 4.1 ± 3.5 (*n* = 38; *p* = 0.02).

**Table 4:** Lesion SUV_max_ parameters (mean ± standard deviation)

SUV parameters	Discovery MI	Gemini TF	*p*
Lesions SUV_max_ (*n* = 68)	8.3 ± 6.5	5.2 ± 4.5	< 0.001*
SUV_max_ in lesions < 1 cm (*n* = 32)	6.5 ± 3.5	3.5 ± 1.7	< 0.001*
SUV_max_ in lesions ≥1 cm (*n* = 36)	9.9 ± 8.1	6.6 ± 5.6	< 0.001*
Lesion-to-blood-pool SUV ratio	4.9 ± 4.0	3.7 ± 3.4	=0.001*
SUV ratio in lesions < 1 cm	3.9 ± 2.2	2.4 ± 1.6	< 0.001*
SUV ratio in lesions ≥1 cm	5.9 ± 5.0	4.9 ± 4.2	=0.05

*Statistically significant based on the Bonferroni adjustment (*p* < 0.008)

#### Image interpretation

**Fig. 3:**
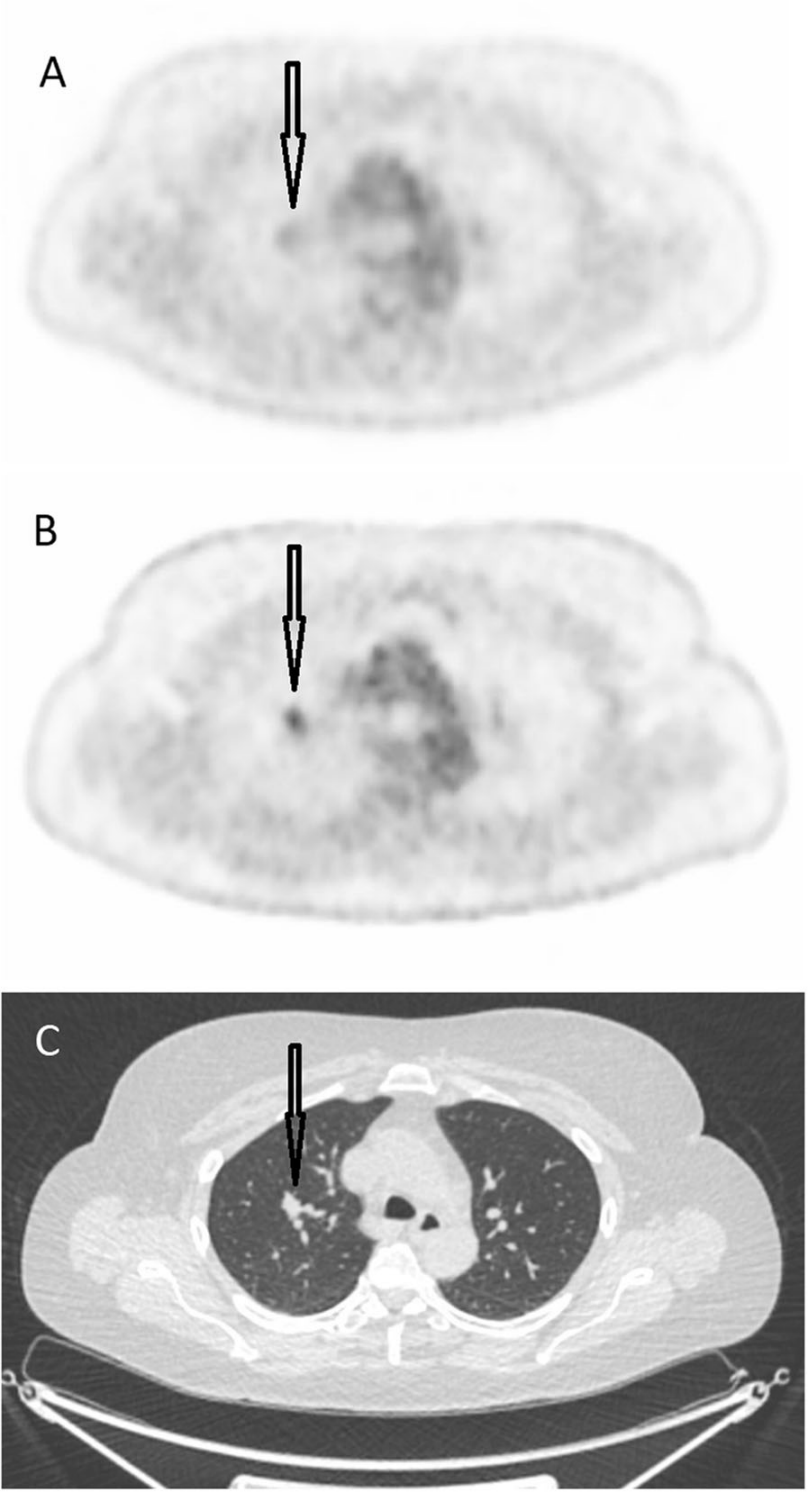
**a**) A transversal image from the Gemini TF. **b**) The same image from the Discovery MI and **c**) the corresponding CT image. The arrow indicates a tumour in the right lung, which was interpreted as malignant in **b**

**Fig. 4:**
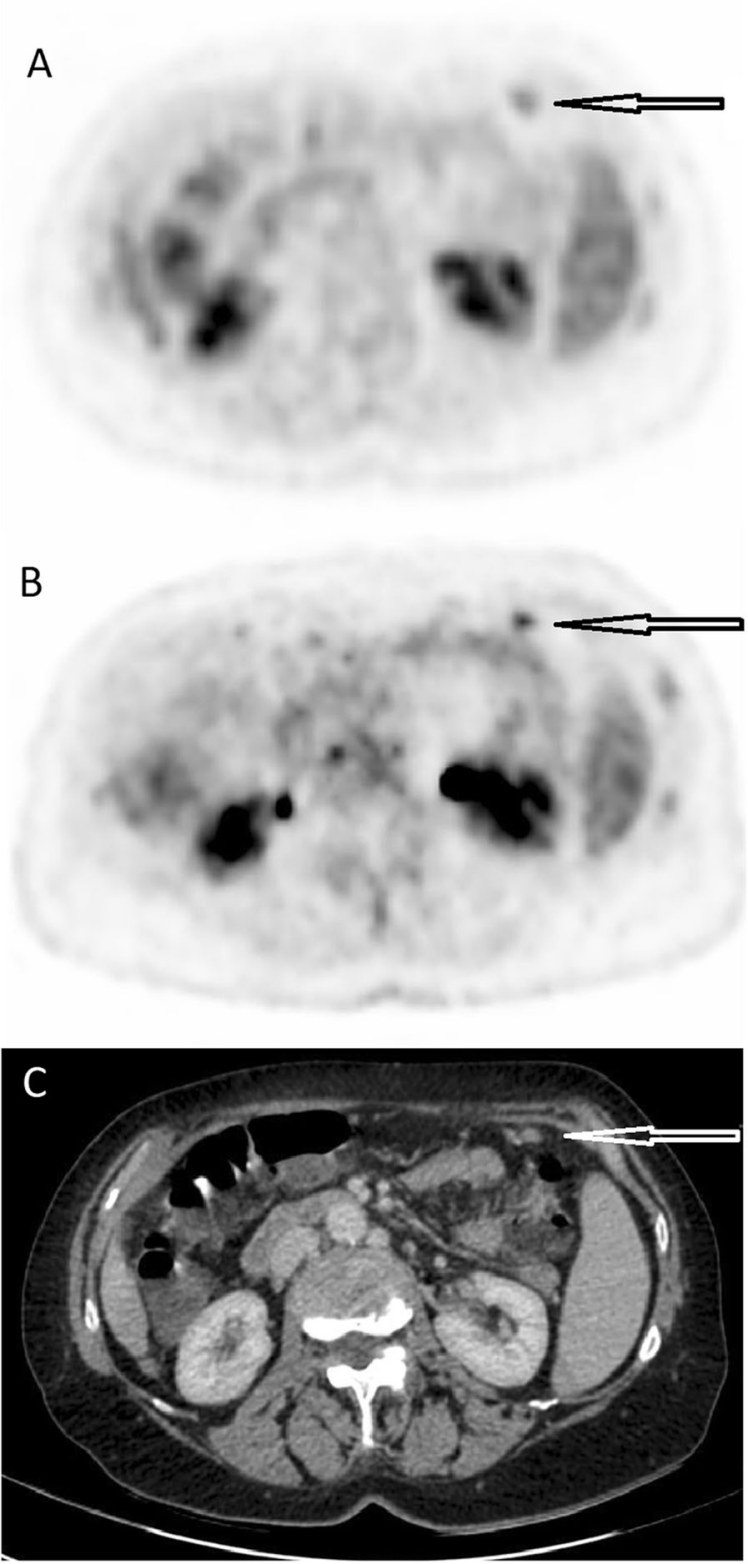
**a**) A transversal image from the Gemini TF. **b**) The same image from the Discovery MI and **c**) the corresponding CT image. The arrow indicates carcinomatosis, more clearly visible in **b**

The total number of malignant/suspected malignant lesions was 32 for images obtained from the Discovery MI and 24 for images obtained from the Gemini TF (*p* = 0.16). The number of inflammatory/suspected inflammatory lesions was 55 for images obtained from the Discovery MI and 29 for images obtained from the Gemini TF (*p* = 0.09). The number of lesions of unclear significance was 38 for the Discovery MI and 24 for the Gemini TF (*p* = 0.27). Table 5 shows the number of lesions found for each patient. Metabolic TNM classification was performed for 15 patients (not for the patient with sarcoidosis). The presence/absence of primary tumour (T) differed in one of the patients (“present” for images from the Discovery MI and “uncertain” for images from the Gemini TF). The presence/absence of spread to lymph nodes (N) was different in one patient (“absent” for images from the Gemini TF and “uncertain” for images from the Discovery MI). The presence/absence of distant metastases (M) was different in two patients (“absent” for images from the Gemini TF and “present” for images from the Discovery MI for both patients). For all other patients, T, N and M classifications were identical for both image sets. Overall, metabolic TNM classification differed in four patients (27%; 95% CI, 10–52%) between the two systems. Table 6 shows the details of the metabolic TNM classifications. Figure 3 shows Patient 14, who was interpreted as having a primary tumour in the right lung when images from the Discovery MI were interpreted (T+), but not when images from the Gemini TF were interpreted (T-). Figure 4 shows Patient 9, where the hyper-metabolism in carcinomatosis is seen more clearly in the Discovery MI images compared to the Gemini TF images.

**Table 5:** Number of lesions for various patients

Patient	Malignant		Inflammatory		Unclear	
	Discovery MI	Gemini TF	Discovery MI	Gemini TF	Discovery MI	Gemini TF
1	3	1	13	0	13	13
2	1	1	7	8	16	4
3	0	0	0	0	0	0
4	1	1	4	3	0	1
5	2	1	0	0	2	2
6	2	2	3	0	0	0
7	0	0	17	11	0	0
8	0	0	0	0	2	1
9	16	11	1	2	0	0
10	2	3	0	0	1	0
11	2	2	0	1	1	1
12	0	0	Multiple	Multiple	0	0
13	0	0	3	0	0	0
14	1	0	1	1	0	1
15	1	1	2	2	0	0
16	1	1	4	1	3	1
Sum	32	24	55	29	38	24

**Table 6:** Presence (+) or absence (−) of primary tumour (T), lymph node metastases (N) and distant metastases (M)

Patient	Discovery MI			Gemini TF		
	T+/−	N+/−	M+/−	T+/−	N+/−	M+/−
1	+	?	**+**	+	?	**–**
2	+	**?**	?	+	**–**	?
3	–	–	–	–	–	–
4	+	–	–	+	–	–
5	+	?	**+**	+	?	**–**
6	–	–	+	–	–	+
7	–	–	–	–	–	–
8	–	–	–	–	–	–
9	+	–	+	+	–	+
10	+	+	–	+	+	–
11	+	–	–	+	–	–
12	N/A	N/A	N/A	N/A	N/A	N/A
13	–	–	–	–	–	–
14	**+**	–	–	**?**	–	–
15	+	–	–	+	–	–
16	+	–	–	+	–	–

## Discussion

The primary finding of this study was that metabolic TNM classifications indicated tumours were at a worse stage (presence of primary tumour, spread to lymph nodes or distant metastases) for images obtained from the Discovery MI compared to those obtained from the Gemini TF in 27% of patients. Also, a trend toward a greater number of malignant, inflammatory and unclear lesions was found with images obtained with the Discovery MI compared to the Gemini TF (although not statistically significant). Also, lesion-to-blood-pool SUV ratios were significantly greater for Discovery MI images compared to Gemini TF images for lesions smaller than 1 cm. Phantom studies showed better image quality for the Discovery MI.

It is clear from analyses of patient lesions and from the phantom analysis that lesions, especially smaller ones, have higher SUVs and are more visible on images obtained from the Discovery MI compared to those from the Gemini TF. However, SUV measurements can be influenced by many factors, including accumulation time [8, 9], patient characteristics and reconstruction method [7, 10–12]. Although SUVs were higher in all but one lesion for images from the Discovery MI, a significantly greater lesion-to-blood-pool SUV ratio was found for lesions smaller than 1 cm. Therefore, the greater SUV is likely explained by the greater sensitivity and different reconstruction algorithm of the Discovery MI system rather than longer accumulation times. The new reconstruction algorithm used with the Discovery MI will decrease the effect of partial volume, particularly for small lesions, due to the point spread function in the Bayesian penalized-likelihood reconstruction algorithm. Using the Gemini TF, this partial volume correction was not possible, leading to a more pronounced underestimation of SUVs in small lesions. Also, the smaller voxel size used by the Discovery MI (2.7 × 2.7 × 2.8 mm) compared to the Gemini TF (4 × 4 × 4 mm) contributes to increased SUVs found using the Discovery MI [13]. A different SUV could also be obtained when using the Q. Clear algorithm because the noise-penalizing determining factor (beta) affects SUV. Thus, our findings depend on both hardware (different generation of PET-CT cameras) and software (different reconstruction methods). The aim of this study was to compare the clinical performances of the two systems. Therefore, the clinical protocols recommended by the vendors were used rather than the same reconstruction method for both.

The greater sensitivity of the Discovery MI permitted shorter times per bed position (1.5 min compared to 2 min for the Gemini TF) with preserved SNRs in the liver for a given dose (4 MBq/kg). The greater axial field of view (20 cm) and the short overlap (24%) permit faster image acquisition. Generally, our experience is that the total PET acquisition time is half that for the Gemini TF. A short acquisition time potentially reduces problems with patient motion and bladder filling and would allow more patients to be examined per day.

It is hoped that the greater sensitivity of SiPM PET-CT and improved reconstruction algorithms will lead to increased detection of pathology, such as earlier detection of small metastases. We found that 27% of patients had different (worse) metabolic TNM classifications using images acquired from the Discovery MI compared to those acquired from the Gemini TF. Unfortunately, we do not know the patients’ true TNM classifications, but our results indicate that more primary tumours, lymph nodes or distant metastases can be detected using the new generation of PET-CT scanners. This agrees with our findings that more malignant and inflammatory lesions were detected (although the difference was not statistically significant, probably because of the limited number of patients included). Also, a greater number of uncertain lesions were found using the Discovery MI PET-CT system. This could be because the physicians interpreting the images were more familiar with images from the Gemini TF, but it could also be a result of the greater number of hypermetabolic lesions found using the novel PET systems. In subsequent studies, where a gold standard is available, it is important to assess both sensitivity and specificity for various diseases using the new generation of PET-CT.

Previous studies of SiPM-based PET platforms in patients have been published. Hsu et al. [4] used the same SiPM-based PET platform as in our study. They presented performance studies of the Discovery MI system and also included a patient case, comparing with a previous-generation PET-CT. The patient, who had melanoma, was scanned using a Discovery 690 PET-CT system (GE Healthcare) which was immediately followed by imaging using the Discovery MI. Several lesions visible only in the Discovery MI images were found, but no gold standard was used to assess true pathology. Nguyen et al. [6] compared the diagnostic performance of a SiPM-based PET prototype scanner with a conventional PM PET scanner using 21 patients who underwent clinical ^18^F-FDG PET-CT. Use of a Gemini TF was followed by use of the SiPM-based prototype, showing better overall image quality with the latter. Lesion SUV_max_ and lesion-to-blood-pool SUV ratios were significantly greater using the SiPM PET compared to conventional PET, and more so for lesions smaller than 1.5 cm. Baratto et al. [5] scanned 50 patients using a conventional PM-based PET-CT followed by a SiPM-based PET-CT, finding that using the latter, more lesions were found as well as higher values for SUV_max_, lesion-to-blood-pool SUV ratio and liver SUV ratio. The findings of these studies agree with our findings. In all these studies, images were acquired using the conventional PM-based PET-CT first. In our study, we attempted to eliminate differences in accumulation time on the two camera systems by scanning about half the patients first using the Gemini TF PET-CT and half in the reverse order.

Additional studies are needed to establish the full potential value of the new generation of PET-CT scanners, but the results from our study, as well as previous studies, indicate that the increased lesion uptake in the new generation of PET-CT, thanks to improved hardware and reconstruction algorithms, can improve diagnostic performance. It is hoped that smaller lesions can be detected and that this will increase sensitivity and specificity for diagnosing various diseases, potentially improving patient management and outcomes.

### Limitations

This study should be viewed in light of some limitations. First, a limited number of patients were included. Second, no gold standard, such as biopsy, or follow-up were performed so that the true number of lesions or true TNM classifications could be assessed. Third, some patients received a diagnostic CT (with intravenous contrast) for one of the PET-CT examinations, whereas others received a low-dose CT. The presence of intravenous contrast is known to slightly affect SUV [14]. Fourth, because the group who received CTs with the Gemini TF first was larger than the group receiving CTs with the Discovery MI first (by two patients), a significantly shorter accumulation time was seen in images obtained from the Gemini TF, which affects SUV and possibly image interpretation. Fifth, no correction for respiratory movement was applied.

## Conclusions

In conclusion, TNM classification was worse in 4 of the 15 patients evaluated for TNM classification, and a trend toward more malignant, inflammatory and unclear lesions was found with images acquired with the Discovery MI compared to the Gemini TF. Also, lesion-to-blood-pool SUV ratios were significantly higher for the Discovery MI compared to the Gemini TF for lesions smaller than 1 cm. It was possible to use a shorter acquisition time for the Discovery MI with preserved SNRs in the liver. Better image quality was found in phantom. Although no gold standard was available, results indicate that the new generation of PET-CT scanners might provide better diagnostic performance, though further studies are needed.

## Data Availability

The datasets used and analysed during the current study are available from the corresponding author on reasonable request.

## Abbreviations

CT: Computed tomography
FDG: Fluorodeoxyglucose
NEMA: National Electrical Manufacturers Association
PET: Positron emission tomography
PM: Photomultiplier
RC: Recovery coefficient
ROI: Region of interest
SiPM: Silicon photomultiplier
SNR: Signal-to-noise
SUV: Standardized uptake value
SUV_max_: maximum standardized uptake value
SUV_mean_: mean standardized uptake value
